# Quantification of serine protease HtrA molecules secreted by the foodborne pathogen *Campylobacter jejuni*

**DOI:** 10.1186/s13099-019-0295-8

**Published:** 2019-04-12

**Authors:** Matthias Neddermann, Steffen Backert

**Affiliations:** Division of Microbiology, Department of Biology, Friedrich Alexander University Erlangen, Staudtstrasse 5, 91058 Erlangen, Germany

**Keywords:** *Campylobacter jejuni*, Serine protease HtrA, Signaling, Secretion, CiaB

## Abstract

**Background:**

*Campylobacter jejuni* is a major food-borne pathogen and a worldwide health threat. Utilizing different virulence factors, *C. jejuni* invades the host’s intestinal epithelial cell layer. One important factor in this process is the serine protease HtrA, which is secreted into the extracellular space, and helps the bacteria to transmigrate across the gut epithelium by cleaving various cell–cell adhesion proteins. The aim of the present study is to quantify the amount of HtrA molecules secreted per bacterial cell in liquid culture and during infection.

**Results:**

HtrA protein purification and quantitative Western blotting were used to determine the number of HtrA molecules secreted by two *C. jejuni* model strains, 11168 and 81-176, in liquid culture during an 8-h time course. On average, the two strains yielded similar HtrA secretion rates, with strain 11168 secreting 4314 ± 949 molecules and 81-176 secreting 5483 ± 1246 per bacterium after 2 h. After 8 h, both strains showed a decrease in the average amount of HtrA secreted per bacterial cell over time. Secretion of HtrA by strain 11168 reduced to about 1772 ± 520 molecules and only 2151 ± 562 HtrA molecules were secreted by strain 81-176 at this time point. During infection of gut epithelial cells, the secretion of HtrA is slightly higher with a similar secretion pattern over time compared to culturing in vitro.

**Conclusion:**

We determined the number of HtrA molecules secreted by single *C. jejuni* cells over time. The results suggest that HtrA secretion is regulated in a time-dependent fashion, leading to increasing accumulative HtrA concentrations in the extracellular medium.

**Electronic supplementary material:**

The online version of this article (10.1186/s13099-019-0295-8) contains supplementary material, which is available to authorized users.

## Background

Food-borne bacteria and their associated diseases are emerging threats in countries all over the world. One major player associated with food-borne diseases is the zoonotic pathogen *Campylobacter jejuni* [1]. As *C. jejuni* needs a habitat with low oxygen concentration it can be found in the intestine of a wide range of wild and domestic birds as well as mammals [2, 3]. Using this route, humans can be infected with *C. jejuni* by consuming contaminated poultry meat, raw milk or other food products [4]. Over 200,000 cases of *C. jejuni* in humans are reported only in the EU per year and the number of unreported cases is estimated to be in the millions [5]. The symptoms of a *C. jejuni* infection typically include fever, stomach cramps or even bloody diarrhea [6]. In rare cases an infection can also lead to chronic diseases like the Miller-Fisher and Guillain-Barrè syndromes [7, 8].

*Campylobacter jejuni* virulence is based on its ability to adhere to and invade effectively into the host’s intestinal epithelial cells. This is enabled by various outer membrane adhesion factors, including MOMP (Major outer membrane protein) and CadF (*Campylobacter* adhesion to fibronectin) [9]. The flagellum of *C. jejuni* is also crucial for the infection pathway, as it is responsible for motility and helps the bacteria to invade into the host cell [10]. Additionally, the flagellum was identified to serve as a type III secretion system (T3SS), which can deliver different proteins into the extracellular milieu or even inject some of them into the host cell to support pathogenicity-associated processes [11–15]. One factor secreted through this T3SS is the *Campylobacter* invasion antigen B (CiaB), which is a 73-kDa protein proposed to be involved in the invasion process of *C. jejuni* [12]. Moreover, some other proteins, including virulence factors, are secreted as cargo in outer membrane vesicles (OMVs), which are continuously shed by the bacteria [16]. For *C. jejuni,* 185 proteins were found to be delivered into the environment by OMVs [17]. However, these proteins were only analyzed in a qualitative manner, while none of them has been quantified yet.

Another important virulence factor of *C. jejuni* is the serine protease HtrA (high temperature requirement A) [18–21]. HtrA proteins are highly conserved in many different pathogens and are crucial for survival during stress conditions [22, 23]. For example, in *C. jejuni* HtrA plays an important role in heat tolerance and oxygen resistance [21]. Most HtrA orthologs consist of a signal peptide, a trypsin-like protease domain, and one or two PDZ domains for protein–protein interaction [24]. The HtrA proteins are usually located in the periplasm, where they act as proteases and chaperones, but *C. jejuni* and its close relative *Helicobacter pylori* are able to secrete HtrA actively into the extracellular space [19, 25, 26], in a manner independent of the flagellum [27]. It appears that secreted HtrA helps the bacteria to transmigrate across the epithelial cell layer in the gut by cleaving the surface adhesion protein E-cadherin, which can at least temporarily open cell-to-cell junctions [19, 26]. It was shown that deletion of the *htrA* gene leads to reduced E-cadherin shedding and to impaired transmigration of *C. jejuni* across polarized epithelial cells in vitro [19]. Further, HtrA is involved in immunopathology and apoptotic responses in the intestine during infection of mice in vivo [28, 29]. All these findings document a role of HtrA for the pathology associated with *C. jejuni* infection. However, the exact secretion mechanism of HtrA is as yet not fully understood. It remained also fully unknown how many HtrA molecules can be secreted by the bacteria during culturing and infection. Here, we quantified the amount of HtrA molecules secreted over time as calculated per single bacterial cell. This represents the first quantification report of a virulence factor secreted by *C. jejuni*. The presented data provide new insights into the secretion efficiency of HtrA, which is a key step in the virulence pathway of *C. jejuni*.

## Results

### Investigation of HtrA secretion in liquid culture by quantitative Western blotting

**Fig. 1:**
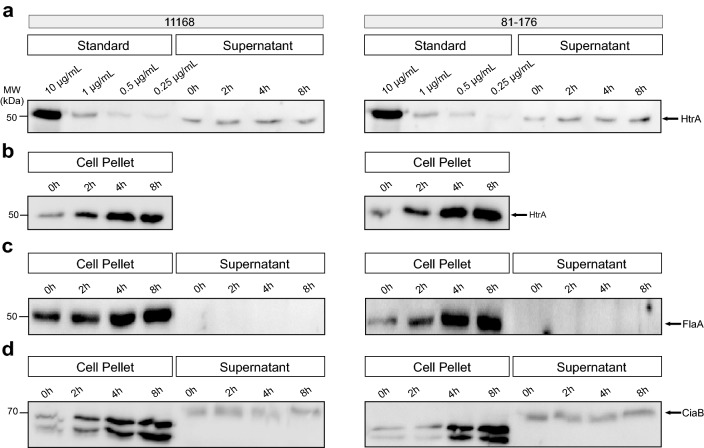
Western blot analysis of the *C. jejuni* 11168 and 81-176 cell pellet and supernatant samples as basis for the quantification. **a** Standard of recombinant HtrA in different dilutions from 10 to 0.25 µg/mL was blotted together with the *C. jejuni* supernatant samples and subjected with antibodies against *C. jejuni* HtrA. Representative supernatant samples are shown of a *C. jejuni* culture taken at 0, 2, 4 and 8 h (right). **b** Corresponding *C. jejuni* pellet samples were also stained with α-HtrA antibodies. **c** Pellet and supernatant samples probed with α-FlaA antibodies as a negative control. **d** Pellet and supernatant samples stained with α-CiaB antibodies as a positive control for secreted proteins. We noted that CiaB in the extracellular fraction migrates at a slightly higher molecular weight (arrow), probably caused by a yet unknown posttranslational modification

The amount of HtrA secreted over time was determined utilizing quantitative Western blotting. First, we investigated HtrA secretion in liquid medium in vitro. For this purpose, samples from two *C. jejuni* model strains (11168 and 81-176) were cultured in BHI broth and analyzed at different time points (0, 2, 4 and 8 h). The yields were compared with a concentration range of purified recombinant HtrA as control. Immunoblots visualized by α-HtrA and control antibodies are displayed for both strains (Fig. 1). Figure 1a shows the standard dilutions of purified recombinant HtrA together with the supernatant samples produced by the different strains. As a control, the corresponding cell pellet samples were blotted separately (Fig. 1b).

Additionally, control blots were stained with anti-flagellin (α-FlaA) antibodies, to exclude the presence of cell debris in the samples (Fig. 1c). For both strains, FlaA signals were only detected in the pellet as expected, while no flagellin was found in the supernatant. As a positive control for secreted proteins, the blots were also stained with α-CiaB antibodies (Fig. 1d). As expected, the results show that secreted CiaB was present both in the pellet and in increasing amounts in the supernatant over time, which illustrates that active protein secretion occurs by both *C. jejuni* strains under our experimental settings.

**Fig. 2:**
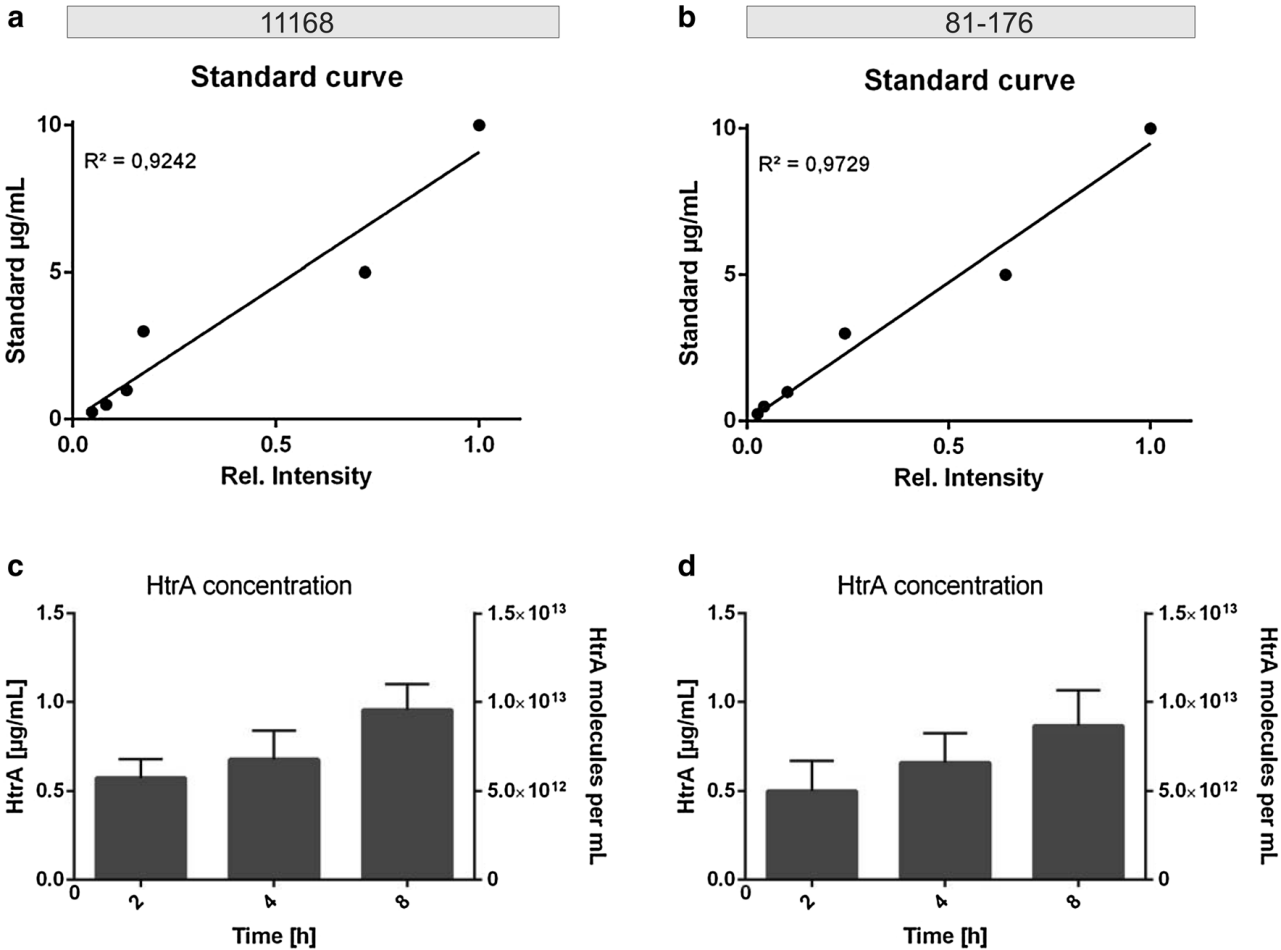
Standard curve of recombinant HtrA and quantification of secreted HtrA in the supernatant of the *C. jejuni* 11168 and 81-176 cultures. **a**, **b** Standard curve for 11168 (**a**) and 81-176 (**b**) of one exemplary experiment based on the band intensities in Western blots against the concentrations of recombinant HtrA. The curve was plotted for each separate experiment based on the corresponding Western blot. **c**, **d** Overview of the secreted HtrA concentrations for 11168 (**c**) and 81-176 (**d**) in µg/mL and as pure numbers for the time points 2, 4 and 8 h. Values were calculated based on the standard curve for each blot separately and the error calculation was done based on the mean of these values (n = 5, error bars = SEM)

Standard curves of recombinant HtrA protein concentrations were produced with a correlation coefficient (R^2^) of 0.9242 for strain 11168 and 0.9729 for 81-176, respectively (Fig. 2). Thus, the quantitative procedure was highly reproducible (Fig. 2a, b), and correlated with the standard depicted in Fig. 1a. Based on this standard curve, the signals obtained from the corresponding culture supernatants were quantified. The concentration of secreted HtrA in the supernatant of both strains increased over time from approximately 0.57 µg/mL for 11168 and around 0.50 µg/mL for strain 81-176 at 2 h to 0.96 µg/mL for 11168 and 0.87 µg/mL for 81-167 at 8 h, respectively (Fig. 2c, d). The extracellular HtrA concentration for both strains reached similar levels and nearly doubled over the 6 h time span. The average results of five independent experiments for both strains are summarized in Tables 1 and 2, respectively. As a control, the amount of intracellular HtrA in the cell pellets was also determined and this increased as the number of bacteria amplified during this growth period, as expected. The molar concentration of secreted HtrA was calculated using its molecular weight of 51,007 (deduced from the amino acid sequence), resulting in approximately 7 × 10^12^ molecules per mL at 2 h, increasing to 11 × 10^12^ molecules at the 8-h time point for 11168 (Fig. 2c, Table 1). Strain 81-176 yielded around 6 × 10^12^ molecules per mL at 2 h, increasing to 10 × 10^12^ molecules after 8 h (Fig. 2d, Table 2). Taken together, the results of these experiments suggest that over 8 h of culturing, HtrA is secreted by both *C. jejuni* strains in similar high amounts.

**Table 1:** Quantification of secreted HtrA in a time course (strain 11168)

Time (h)	HtrA concentration (µg/mL)	Molecules of HtrA per mL (× 10^8^)	Bacteria (× 10^9^ CFU/mL)	Molecules HtrA secreted per CFU	Molecules HtrA secreted per CFU (with Caco-2 cells)
t = 2	0.5748 ± 0.1069	67,880 ± 12,630	1.8 ± 0.07	4314 ± 949	8277 ± 1306
t = 4	0.6793 ± 0.1595	80,210 ± 18,840	3.1 ± 0.24	2979 ± 761	4021 ± 637
t = 8	0.9554 ± 0.1458	112,800 ± 17,220	5.0 ± 0.41	1772 ± 520	1658 ± 759

Values were calculated for each experiment separately and then the mean of these values was calculated. The shown error values for the first three columns are SEM and based on the final mean values. The errors for the fourth and fifth column were calculated as described in the “Materials and methods” section

**Table 2:** Quantification of secreted HtrA in a time course (strain 81-176)

Time (h)	HtrA concentration (µg/mL)	Molecules of HtrA per mL (× 10^8^)	Bacteria (× 10^9^ CFU/mL)	Molecules HtrA secreted per CFU	Molecules HtrA secreted per CFU (with Caco-2 cells)
t = 2	0.5009 ± 0.1699	59,140 ± 20,070	1.7 ± 0.08	5483 ± 1,246	9640 ± 1078
t = 4	0.6604 ± 0.1623	77,980 ± 19,170	3.0 ± 0.26	3466 ± 814	5279 ± 1655
t = 8	0.8654 ± 0.2006	102,200 ± 23,690	5.1 ± 0.48	2151 ± 562	3263 ± 317

Values were calculated for each experiment separately and then the mean of these values was calculated. The shown error values for the first three columns are SEM and based on the final mean values. The errors for the fourth and fifth column were calculated as described in the “ Materials and methods” section

### Amount of HtrA secreted per bacterium in liquid culture decreases over time

**Fig. 3:**
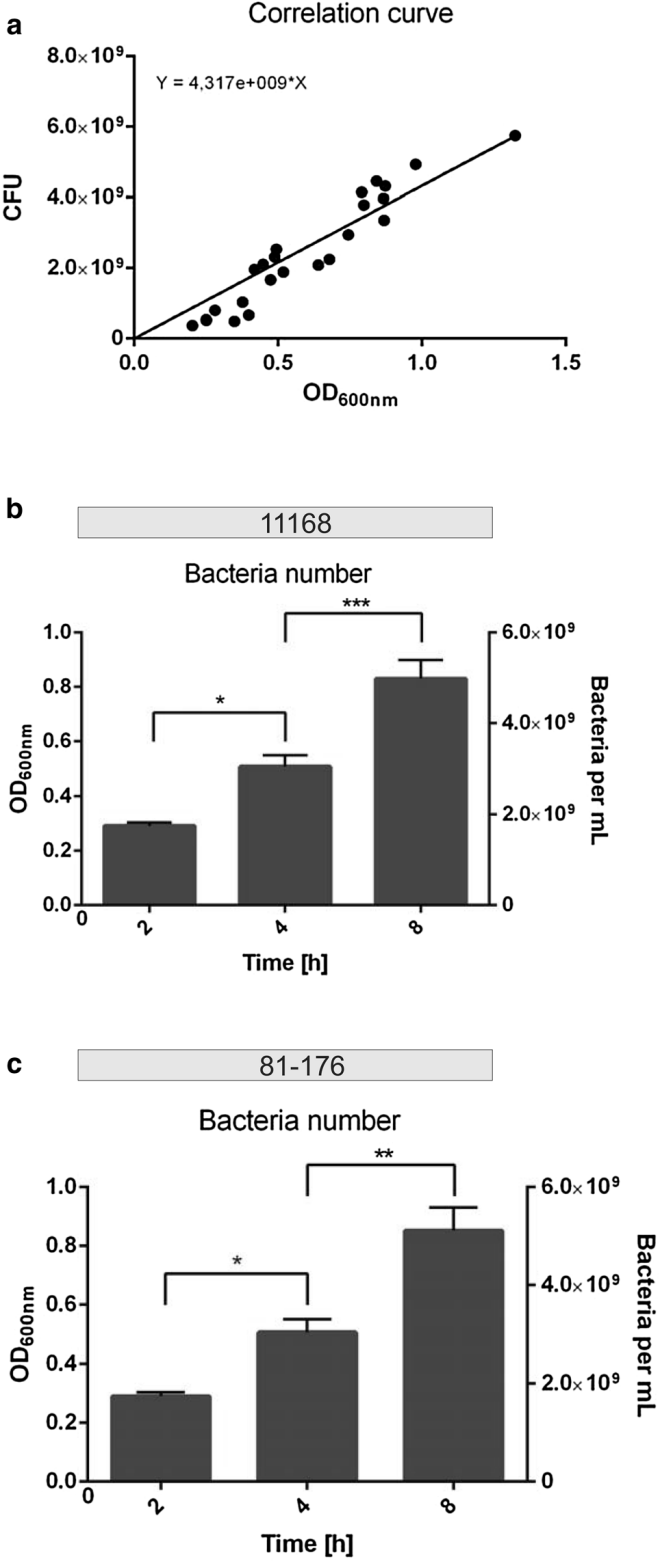
Determining the bacteria number in the liquid culture. **a** Correlation curve for *C. jejuni* wild-type. CFU per mL are plotted against their matching OD_600 nm_ values. **b**, **c** Overview of the bacteria number for each time point for 11168 (**b**) and 81-176 (**c**). The bacteria per mL were calculated by inserting the OD_600 nm_ values into the correlation curve equation (n = 5, error bars = SEM)

**Fig. 4:**
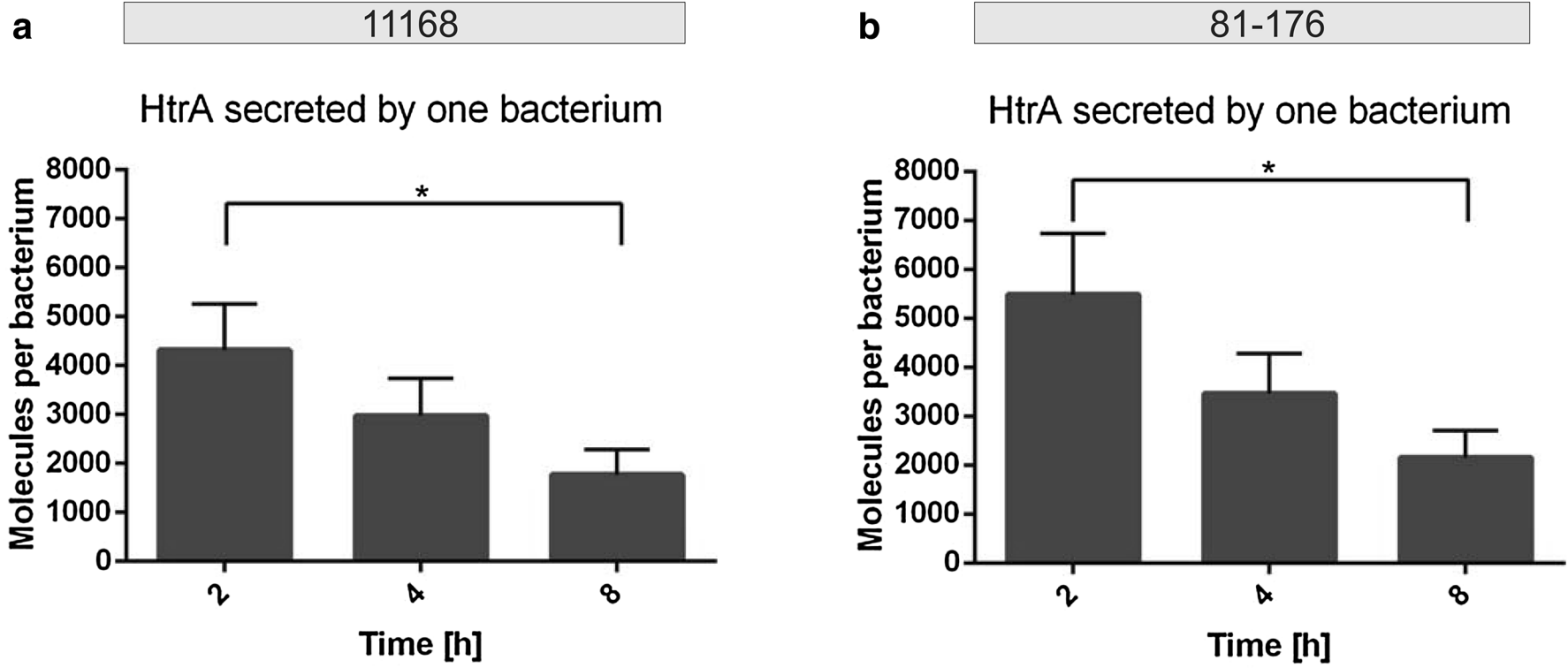
Quantification of secreted HtrA molecules by single bacterial cells in liquid culture. **a**, **b** Average amount of HtrA molecules secreted by one bacterium in liquid culture at each time point of the experiment calculated for 11168 (**a**) and 81-176 (**b**) (n = 5, error bars = SEM)

The exact number of colony-forming units (CFU) of bacteria in liquid cultures of different OD_600 nm_ values was established by serial dilution, and an OD_600 nm_/CFU correlation curve was established (Fig. 3a). This correlation curve is based on multiple independent experimental measurements of both strains, as strain-dependent differences were not observed. During the secretion assay of 11168, bacterial cell numbers more than doubled from approximately 1.8 × 10^9^ CFU/mL at time point 2 h to 5 × 10^9^ CFU at 8 h (Fig. 3b, Table 1). The assay with strain 81-176 yielded a similar result with 1.7 × 10^9^ CFU/mL at 2 h to 5.1 × 10^9^ CFU at 8 h (Fig. 3c, Table 2). These data provided the basis to calculate the average amount of secreted HtrA per single bacterial cell. For strain 11168, the values decreased from around 4300 molecules per cell on average at time point 2 h to nearly 3000 molecules per cell at 4 h and further down to about 1800 molecules per cell at 8 h (Fig. 4a, Table 1). The calculations for strain 81-176 showed a similar trend, starting with nearly 5500 molecules per cell at 2 h, decreasing to ca. 3500 molecules per cell after 4 h and going down to around 2100 molecules on average per cell at 8 h (Fig. 4b, Table 2).

### Presence of human host cells stimulates HtrA secretion

**Fig. 5:**
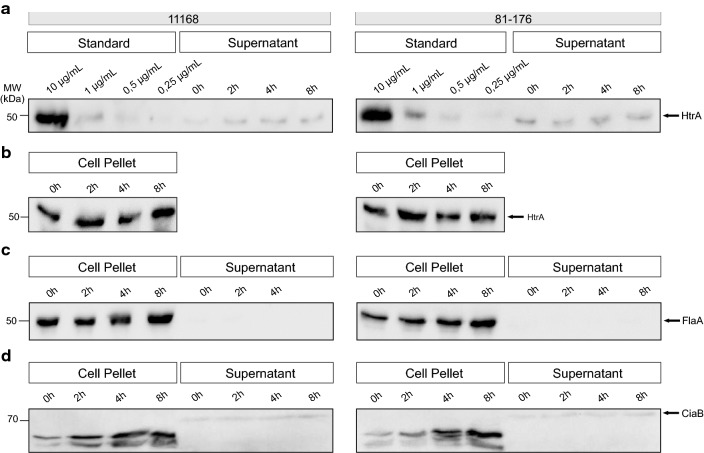
Western blot analysis of HtrA in the *C. jejuni* 11168 and 81-176 pellets and supernatants in the presence of Caco-2 cells. **a** Standard of recombinant HtrA in different dilutions from 10 to 0.25 µg/mL was blotted together with the *C. jejuni* supernatant samples and subjected to immunoblotting with antibodies against *C. jejuni* HtrA. Representative supernatant samples are shown of a *C. jejuni* culture taken at 0, 2, 4 and 8 h (right). **b** Corresponding *C. jejuni* pellet samples were also stained with α-HtrA antibodies. **c** Pellet and supernatant samples probed with α-FlaA antibodies as a negative control. **d** Pellet and supernatant samples stained with α-CiaB antibodies as a positive control for secreted proteins. We noted that CiaB in the extracellular fraction migrates at a slightly higher molecular weight (arrow), probably caused by a yet unknown posttranslational modification

**Fig. 6:**
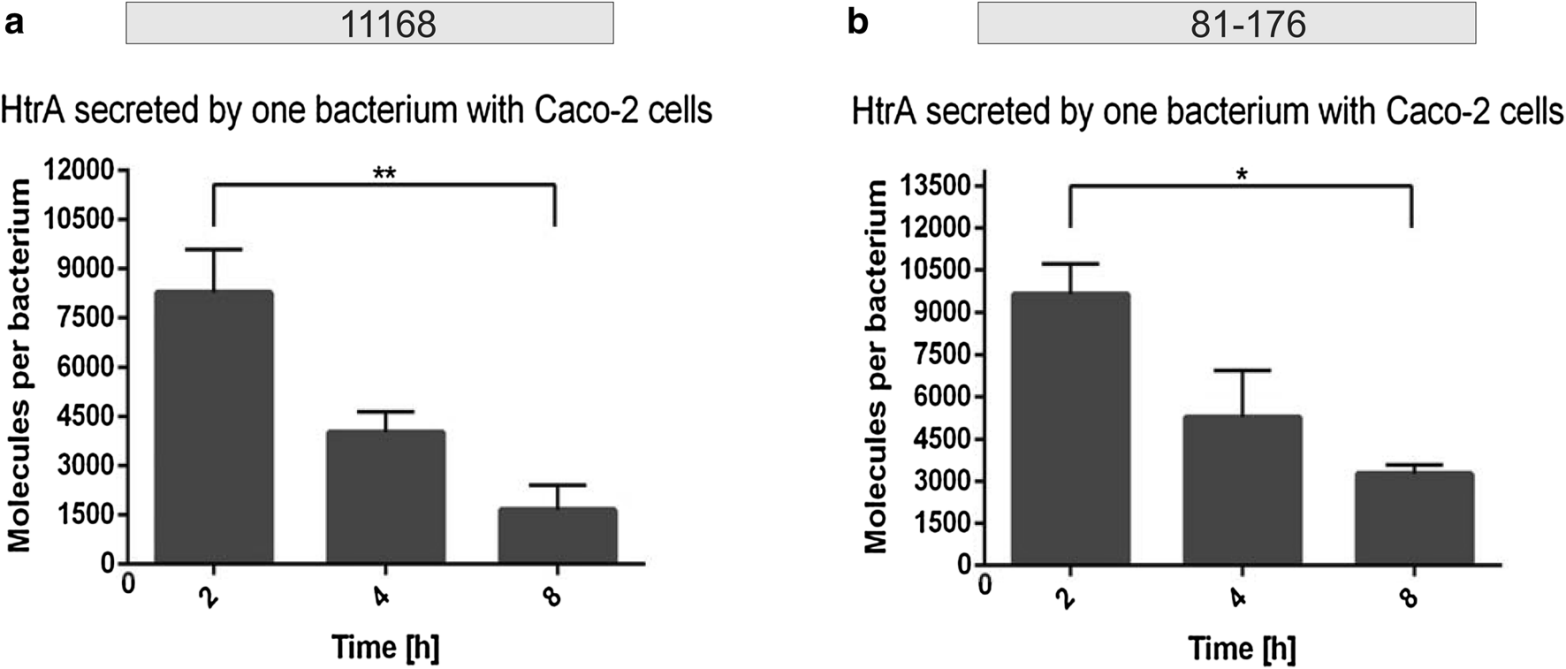
Quantification of secreted HtrA molecules by single *C. jejuni* 11168 and 81-176 cells in the presence of Caco-2 cells. **a**, **b** Average amount of HtrA molecules secreted by single bacterial cells at each time point of the experiment was calculated for 11168 (**a**) and 81-176 (**b**) (n = 3, error bars = SEM)

Finally, we investigated if HtrA secretion may be influenced in the presence of human host target cells. For this purpose, cultured Caco-2 intestinal epithelial cells were infected with either *C. jejuni* strain. Samples were collected at three different time points and subjected to Western blotting (Fig. 5). The quantification of the average number of HtrA molecules secreted by one bacterial cell in the presence of host cells revealed a decrease for both strains over time. Strain 11168 secreted around 8000 HtrA molecules per bacterial cell after 2 h, 4000 molecules at time point 4 h and nearly 1700 molecules per single bacterial cell after 8 h of incubation in presence with Caco-2 cells (Fig. 6a, Table 1). For strain 81-176, the secretion of HtrA molecules per single bacterial cell were above 9600 in average after 2 h, around 5300 molecules per bacterial cell after 4 h and went down to about 3200 molecules at time point 8 h (Fig. 6b, Table 2). Thus, the decrease of HtrA secreted per bacterial cell in average over time is similar to the findings with liquid culture, but the presence of the Caco-2 cells seems to stimulate the secretion of HtrA at the beginning of the infection.

Taken together, our data show that for both examined strains of *C. jejuni* the average amount of HtrA secreted by one bacterial cell can be very high, but decreases over time. This leads to a model where each *C. jejuni* cell is secreting HtrA in vast amounts during early log phase, but the rate of HtrA secretion slows down over time, while the accumulative amount of HtrA molecules in the medium continues to increase throughout the investigated time period of 8 h.

## Discussion

The serine protease HtrA is an important virulence factor of *C. jejuni* and also plays an prominent role in various cellular processes [18–21]. During infection, HtrA is secreted into the extracellular environment, where it enables *C. jejuni* to transmigrate through the paracellular space of the host’s gut epithelial cells by cleavage of cell adhesion proteins such as E-cadherin [19, 26]. Although HtrA is an important pathogenicity determinant of *C. jejuni*, the amount of HtrA molecules secreted by the bacteria has never been determined. Therefore, in this study, we quantified the average number of HtrA molecules secreted per bacterial cell in a time course. This is the first time a secreted virulence factor of *C. jejuni* has been quantified. Our assay detects secreted proteins by means of quantitative Western blotting. Previously, we successfully used a similar approach to quantify the secretion of HtrA by *Helicobacter pylori* [30].

The quantification assay is based on utilizing a standard curve [31], that in our case was based on recombinant *C. jejuni* HtrA. It is essential that the used recombinant protein is clean and its concentration is precisely determined, which has been ensured in the present study. Since equal volumes of the standard and the samples are loaded on the same SDS-PAGE gel and then visualized with the same antibody, there is a direct correlation between the detected band intensities and the loaded protein concentrations. Furthermore, the reproducibility of the experiments is very important. The liquid culture experiments were repeated at least five times and the infection experiments at least three times for each strain and these independent replicates were performed under identical conditions. The obtained data for the cell counts and HtrA concentrations revealed acceptable error margins, which were appropriately considered in the calculations. Hence, the here described experimental setup enables to accurately track how much of a single virulence factor is secreted by *C. jejuni* and can be applied to secreted virulence factors of any other bacteria.

Quantifying the average amount of HtrA molecules secreted per single *C. jejuni* cell provides a wider understanding of the secretion mechanism of this protease. The data presented here, obtained for the two strains 81-176 and 11168, imply that the number of HtrA molecules secreted by one bacterial cell is first increased and then decreasing over time, while the overall amount of secreted HtrA is accumulative. This suggests a model in which each bacterial cell first secretes a large amount of HtrA molecules and then downregulates the secretion over time when the bacterial numbers rise. In the presence of eukaryotic host cells, this effect is even increased. At the beginning of the infection, the bacteria secrete more HtrA molecules than in the liquid broth culture, which suggest that the secretion of HtrA is stimulated through presence of the host cells by a yet unknown mechanism. The overall concentration then decreases over time in a similar manner compared to the liquid culture secretion. One possible explanation for the decrease of HtrA over time is that the bacteria may sense the concentration of extracellular HtrA molecules by a yet unknown mechanism and downregulate the secretion accordingly. Another plausible scenario could be autodegradation of the HtrA molecules in solution. In fact, autocleavage of HtrA has been described in *E. coli* [32] and *H. pylori* [33]. However, specific degradation products were not detected by our polyclonal HtrA antibodies on Western blots, but autodegradation in *C. jejuni* cannot be excluded. Finally, a possible explanation for the even lower HtrA levels detected in the presence of host cells is that a substantial number of bacteria and HtrA molecules could attach to the epithelium and cannot be detected any longer in the supernatant [15]. Together, whether one or the other option applies to *C. jejuni* HtrA requires further studies. Additionally, this differs from *H.* *pylori*, which showed a constant HtrA secretion pattern both in liquid culture and in the presence of host cells [30].

The data obtained in this study not only improve our understanding of the HtrA secretion by *C. jejuni*, but may also assist in working towards the development of an effective HtrA inhibitor, because when the precise number of HtrA molecules in a bacterial culture is identified, the dose of the inhibitor can be adjusted accordingly. Such an inhibitor would be useful to combat various bacterial pathogens that secrete HtrA, including *H. pylori* [25, 34], *Borrelia burgdorferi* [35, 36] and *Chlamydia* species [37, 38]. In particular, different *H. pylori* HtrA inhibitors are currently under development in order to inhibit E-cadherin cleavage [39, 40]. Understanding the dynamics of HtrA secretion in these different bacteria could help working towards effective and pathogen-specific inhibitors. Additionally, the here presented assay can be used to quantify other secreted virulence factors of *C. jejuni*.

## Conclusions

Taken together, the assay described here enables for the first time to quantify a virulence factor secreted by *C. jejuni*, in this case the serine protease HtrA. The two investigated strains, 81-176 and 11168, display a similar secretion pattern, where the average number of HtrA molecules secreted per bacterial cell is decreasing over time, whereby in a growing culture the accumulative amount of HtrA in the solution is increasing, while the secretion per individual bacterial cell decreases over time. During infection experiments with eukaryotic cells the secretion of HtrA is initially increased, but then also decreases over time.

## Materials and methods

### *C. jejuni* and *E. coli* growth

*Campylobacter jejuni* strains 81-176 and NCTC11168 were grown on *Campylobacter* blood-free selective Agar Base (Oxoid, Wesel, Germany) plates containing *Campylobacter* supplement (Oxoid) for 2 days at 37 °C in an anaerobic jar together with a CampyGen gas mix (Oxoid). For CFU counting the bacteria were plated on Mueller–Hinton (MH) agar (Oxoid) with 10 µg/mL vancomycin. *E. coli* BL21 (NEB, Ipswitch, USA) was cultivated in Terrific Broth (TB) medium containing 20 g/L tryptone, 24 g/L yeast extract, 4 mL/L glycerol, 0.072 M K_2_HPO_4_ and 0.017 M KH_2_PO_4_ at 37 °C under agitation.

### Overexpression and purification of HtrA

*Escherichia coli* BL21 transformed with expression vector pGEX-6P-1 containing the *htrA* gene of *C. jejuni* strain 81-176 fused to an amino-terminal GST-tag was grown in TB-medium in the presence of 100 µg/mL ampicillin [26]. The overexpression was induced by 0.1 mM isopropyl-β-d-thiogalactopyranoside (IPTG) as previously described [19]. The culture was harvested by centrifugation at 5000×*g* and 4 °C for 15 min and the bacteria were lysed in buffer A (50 mM Tris–HCl, 150 mM NaCl, 1 mM EDTA, 1 mM DTT, pH 8) by sonication. The lysate was then centrifuged (15,000×*g* at 4 °C, 1 h) and the supernatant was filtered through a 0.25 µM filter (Sarstedt, Nümbrecht, Germany) and loaded on a prepacked 5 mL GSTrap-column (GE Healthcare, Buckinghamshire, UK), which was equilibrated with buffer A. After elution using buffer B (50 mM Tris–HCl, 150 mM NaCl, 1 mM DTT, 1 mM EDTA, 5 mM glutathion, pH 8), 10 U PreScission protease (GE Healthcare) were added and incubated for 3 h at 4 °C to cut of the GST-tag. For the final purification step, to separate the protein and the cut-off tag, the solution was loaded on a Superdex-75 gel filtration column (GE Healthcare) and eluted in HEPES buffer (pH 7.4). The purity of the eluted protein was checked by SDS-PAGE and Coomassie Brilliant Blue staining (Bio-Rad, Munich, Germany) and was found to be of > 95% purity. A Coomassie stained acrylamide gel of the purified recombinant protein is shown in Additional file 1: Figure S1.

### HtrA secretion assay and quantitative western blotting

*Campylobacter jejuni* 81-176 or 11168 bacteria were harvested from agar plates and resuspended in prewarmed BHI medium (Oxoid) using sterile cotton swabs (Carl Roth, Karlsruhe/Germany) to OD_600 nm_ = 0.3 to start the HtrA secretion assay. The liquid culture was incubated at 37 °C under shaking (160 rpm) and samples were taken at three different time points (2, 4 and 8 h). The samples were immediately centrifuged (13,000×*g* for 10 min at 4 °C) to collect both the supernatant and the cell pellet. The 8 h time scale was chosen to investigate the HtrA secretion during the log phase of bacterial growth.

The cell pellets were resuspended in BHI medium to their original sample volume. Both the resuspended pellets and the collected supernatants were mixed 3:1 (v/v) with 4× SDS buffer containing 1.5 M Tris–HCl pH 6.8, 20% sodium dodecyl sulfate, 0.05% bromophenol blue, 40% glycerol and 0.05% 2-mercaptoethanol as reducing agent (Thermo Fisher Scientific, Waltham, USA). A concentration range (0.25, 0.5, 1.0 and 10 µg/mL) of purified recombinant HtrA was included as the standard. Prior to electrophoresis using 10% SDS polyacrylamide gels (SDS-PAGE) the samples were denatured at 94 °C for 10 min.

Western blotting was performed onto PVDF membranes (Carl Roth) by standard procedures, after which the membranes were blocked with 5% milk powder in 1.4 M NaCl, 0.2 M Tris, 1% Tween at 4 °C overnight. HtrA was detected by incubation with primary rabbit antibody α-HtrA as previously described [18, 41], incubated for 2 h at room temperature, followed by incubation with horseradish peroxidase-conjugated secondary antibody for 1 h. The membrane was also incubated with previously described α-CiaB antibodies [21] and α-FlaA antibodies as controls (Austral Biologicals, San Ramon/USA). Antibody detection was performed using the Clarity™ Western ECL Kit (Bio-Rad).

Quantitation of HtrA bands was performed using Image Lab software (Bio-Rad), whereby the intensity of the recombinant HtrA standard curve served as a reference. All experiments were performed in five replicates. The concentration of HtrA in µg/mL was converted to molar concentrations using the molecular weight of the protein (51,007).

### HtrA secretion assay in the presence of Caco-2 cells

Human colon adenocarcinoma Caco-2 cells (ATCC HTB-37) were cultured until confluent in 12-well plates in RPMI 1640 medium (Invitrogen, Karlsruhe/Germany) containing 10% FCS (Invitrogen*).* A suspension of either *C. jejuni* strain was added to each well to a final OD_600 nm_ of 0.3 and the plates were further incubated at 37 °C and 5% CO_2_. The bacteria were also added to a control 12-well plate lacking Caco-2 cells but containing the RPMI medium. At three different time points (2, 4 and 8 h) the medium of single wells was harvested and immediately centrifuged (13,000×*g* for 10 min at 5 °C) to collect both the supernatant and the bacteria pellet. Again, this time course was chosen to monitor the HtrA secretion levels during the log phase of bacterial growth. From here on the samples were treated and blotted as described above. Additionally, at each time point a well of the control plate was plated by serial dilution for CFU numeration using standard procedures.

### *Campylobacter jejuni* OD_600 nm_/CFU correlation curve and calculations

Using the same procedure as above for the liquid culture experiments, a *C. jejuni* suspension of OD_600 nm_ = 0.2 was incubated at 160 rpm and 37 °C to determine a correlation curve for conditions that applied during the secretion assay. Hourly samples were taken to measure OD_600 nm_ and serial dilutions were plated for CFU counting and a correlation curve was plotted.

The number of viable bacteria during the secretion assay was calculated based on this correlation curve. The mean concentrations of secreted HtrA and of the bacterial CFUs were used to calculate the number of molecules secreted per cell. The error propagation was calculated using the following equation.

$${\text{U}}c = \sqrt {\left( {\frac{\partial c}{\partial a} \cdot Ua} \right)^{2} + \left( {\frac{\delta c}{\delta b} \cdot Ub} \right)^{2} }$$

where the variable *a* is the molar concentration of secreted HtrA per mL, *b* is the concentration of bacteria (CFU/mL), *c* is the ratio of molecules secreted per colony-forming cell, and U is the error of each of these variables.

### Statistics

All experiments were done at least three times with similar results. All data were evaluated via one-way ANOVA and Bonferroni’s multiple comparison test with GraphPad Prism 6. Statistical significance was defined by *p* ≤ 0.05 (*), *p* ≤ 0.01 (**) and *p* ≤ 0.001 (***). The general errors were calculated as standard error of mean (SEM).

## Additional file

**Additional file 1: Figure S1.** Purification of recombinant *C. jejuni* HtrA expressed in *E. coli* BL21. Important purification steps are shown, including purification by use of a GST-affinity column, exemplary wash and two elution fractions. The final purification step was performed via gel filtration, showing purified HtrA in fraction 2.

## References

[1] Young KT, Davis LM, Dirita VJ (2007). *Campylobacter jejuni*: molecular biology and pathogenesis. Nat Rev Microbiol.

[2] Kim JC, Oh E, Hwang S, Ryu S, Jeon B (2015). Non-selective regulation of peroxide and superoxide resistance genes by PerR in *Campylobacter jejuni*. Front Microbiol.

[3] Ugarte-Ruiz M, Dominguez L, Corcionivoschi N, Wren BW, Dorrell N, Gundogdu O (2018). Exploring the oxidative, antimicrobial and genomic properties of *Campylobacter jejuni* strains isolated from poultry. Res Vet Sci.

[4] Poly F, Guerry P (2008). Pathogenesis of *Campylobacter*. Curr Opin Gastroenterol.

[5] European Food Safety Authority and European Centre for Disease Prevention and Control A (2008). The European Union summary report on antimicrobial resistance in zoonotic and indicator bacteria from humans, animals and food in 2016. EFSA J.

[6] Kist M, Bereswill S (2001). *Campylobacter jejuni*. Contrib Microbiol.

[7] Wakerley BR, Uncini A, Yuki N, Group GBSC, Group GBSC (2014). Guillain-Barre and Miller Fisher syndromes—new diagnostic classification. Nat Rev Neurol.

[8] Talukder RK, Sutradhar SR, Rahman KM, Uddin MJ, Akhter H (2011). Guillian-Barre syndrome. Mymensingh Med J.

[9] Croinin TO, Backert S (2012). Host epithelial cell invasion by *Campylobacter jejuni*: trigger or zipper mechanism?. Front Cell Infect Microbiol.

[10] Szymanski CM, King M, Haardt M, Armstrong GD (1995). *Campylobacter jejuni* motility and invasion of Caco-2 cells. Infect Immun.

[11] Christensen JE, Pacheco SA, Konkel ME (2009). Identification of a *Campylobacter jejuni*-secreted protein required for maximal invasion of host cells. Mol Microbiol.

[12] Konkel ME, Gray SA, Kim BJ, Garvis SG, Yoon J (1999). Identification of the enteropathogens *Campylobacter jejuni* and *Campylobacter coli* based on the cadF virulence gene and its product. J Clin Microbiol.

[13] Neal-McKinney JM, Christensen JE, Konkel ME (2010). Amino-terminal residues dictate the export efficiency of the *Campylobacter jejuni* filament proteins via the flagellum. Mol Microbiol.

[14] Young GM, Schmiel DH, Miller VL (1999). A new pathway for the secretion of virulence factors by bacteria: the flagellar export apparatus functions as a protein-secretion system. Proc Natl Acad Sci USA.

[15] Barrero-Tobon AM, Hendrixson DR (2012). Identification and analysis of flagellar coexpressed determinants (Feds) of *Campylobacter jejuni* involved in colonization. Mol Microbiol.

[16] Jang KS, Sweredoski MJ, Graham RL, Hess S, Clemons WM (2014). Comprehensive proteomic profiling of outer membrane vesicles from *Campylobacter jejuni*. J Proteomics.

[17] Elmi A, Dorey A, Watson E, Jagatia H, Inglis NF, Gundogdu O (2018). The bile salt sodium taurocholate induces *Campylobacter jejuni* outer membrane vesicle production and increases OMV-associated proteolytic activity. Cell Microbiol.

[18] Baek KT, Vegge CS, Brondsted L (2011). HtrA chaperone activity contributes to host cell binding in *Campylobacter jejuni*. Gut Pathog.

[19] Boehm M, Hoy B, Rohde M, Tegtmeyer N, Baek KT, Oyarzabal OA (2012). Rapid paracellular transmigration of *Campylobacter jejuni* across polarized epithelial cells without affecting TER: role of proteolytic-active HtrA cleaving E-cadherin but not fibronectin. Gut Pathog.

[20] Brondsted L, Andersen MT, Parker M, Jorgensen K, Ingmer H (2005). The HtrA protease of *Campylobacter jejuni* is required for heat and oxygen tolerance and for optimal interaction with human epithelial cells. Appl Environ Microbiol.

[21] Boehm M, Lind J, Backert S, Tegtmeyer N (2015). *Campylobacter jejuni* serine protease HtrA plays an important role in heat tolerance, oxygen resistance, host cell adhesion, invasion, and transmigration. Eur J Microbiol Immunol.

[22] Backert S, Bernegger S, Skorko-Glonek J, Wessler S (2018). Extracellular HtrA serine proteases: an emerging new strategy in bacterial pathogenesis. Cell Microbiol.

[23] Skorko-Glonek J, Zurawa-Janicka D, Koper T, Jarzab M, Figaj D, Glaza P (2013). HtrA protease family as therapeutic targets. Curr Pharm Des.

[24] Kim DY, Kim KK (2005). Structure and function of HtrA family proteins, the key players in protein quality control. J Biochem Mol Biol.

[25] Hoy B, Lower M, Weydig C, Carra G, Tegtmeyer N, Geppert T (2010). *Helicobacter pylori* HtrA is a new secreted virulence factor that cleaves E-cadherin to disrupt intercellular adhesion. EMBO Rep.

[26] Hoy B, Geppert T, Boehm M, Reisen F, Plattner P, Gadermaier G (2012). Distinct roles of secreted HtrA proteases from gram-negative pathogens in cleaving the junctional protein and tumor suppressor E-cadherin. J Biol Chem.

[27] Boehm M, Haenel I, Hoy B, Brondsted L, Smith TG, Hoover T (2013). Extracellular secretion of protease HtrA from *Campylobacter jejuni* is highly efficient and independent of its protease activity and flagellum. Eur J Microbiol Immunol.

[28] Heimesaat MM, Fischer A, Alutis M, Grundmann U, Boehm M, Tegtmeyer N (2014). The impact of serine protease HtrA in apoptosis, intestinal immune responses and extra-intestinal histopathology during *Campylobacter jejuni* infection of infant mice. Gut Pathog.

[29] Heimesaat MM, Lugert R, Fischer A, Alutis M, Kuhl AA, Zautner AE (2014). Impact of C*ampylobacter jejuni* cj0268c knockout mutation on intestinal colonization, translocation, and induction of immunopathology in gnotobiotic IL-10 deficient mice. PLoS ONE.

[30] Neddermann M, Backert S. How many protein molecules are secreted by single *Helicobacter pylori* cells: quantification of serine protease HtrA. Cell Microbiol. 2019:e13022. 10.1111/cmi.13022 30822363

[31] Suzuki O, Koura M, Noguchi Y, Uchio-Yamada K, Matsuda J (2011). Use of sample mixtures for standard curve creation in quantitative western blots. Exp Anim.

[32] Jomaa A, Iwanczyk J, Tran J, Ortega J (2009). Characterization of the autocleavage process of the *Escherichia coli* HtrA protein: implications for its physiological role. J Bacteriol.

[33] Albrecht N, Tegtmeyer N, Sticht H, Skorko-Glonek J, Backert S (2018). Amino-terminal processing of *Helicobacter pylori* serine protease HtrA: role in oligomerization and activity regulation. Front Microbiol.

[34] Tegtmeyer N, Moodley Y, Yamaoka Y, Pernitzsch SR, Schmidt V, Traverso FR (2016). Characterisation of worldwide *Helicobacter pylori* strains reveals genetic conservation and essentiality of serine protease HtrA. Mol Microbiol.

[35] Russell TM, Delorey MJ, Johnson BJ (2013). *Borrelia burgdorferi* BbHtrA degrades host ECM proteins and stimulates release of inflammatory cytokines in vitro. Mol Microbiol.

[36] Russell TM, Johnson BJ (2013). Lyme disease spirochaetes possess an aggrecan-binding protease with aggrecanase activity. Mol Microbiol.

[37] Lawrence A, Fraser T, Gillett A, Tyndall JD, Timms P, Polkinghorne A (2016). *Chlamydia* serine protease inhibitor, targeting HtrA, as a new treatment for Koala *Chlamydia* infection. Sci Rep.

[38] Ong VA, Lawrence A, Timms P, Vodstrcil LA, Tabrizi SN, Beagley KW (2015). In vitro susceptibility of recent *Chlamydia trachomatis* clinical isolates to the CtHtrA inhibitor JO146. Microbes Infect.

[39] Perna AM, Rodrigues T, Schmidt TP, Bohm M, Stutz K, Reker D (2015). Fragment-based de novo design reveals a small-molecule inhibitor of *Helicobacter pylori* HtrA. Angew Chem Int Ed Engl.

[40] Schmidt TP, Perna AM, Fugmann T, Bohm M, Jan H, Haller S (2016). Identification of E-cadherin signature motifs functioning as cleavage sites for *Helicobacter pylori* HtrA. Sci Rep.

[41] Baek KT, Vegge CS, Skorko-Glonek J, Brondsted L (2011). Different contributions of HtrA protease and chaperone activities to *Campylobacter jejuni* stress tolerance and physiology. Appl Environ Microbiol.

