# Supplementary figures and images for: Quantification of serine protease HtrA molecules secreted by the foodborne pathogen Campylobacter jejuni
Source: Gut Pathog. 2019 Apr 12;11:14. doi: 10.1186/s13099-019-0295-8 (PMC6460743; doi:10.1186/s13099-019-0295-8)

Figure S1

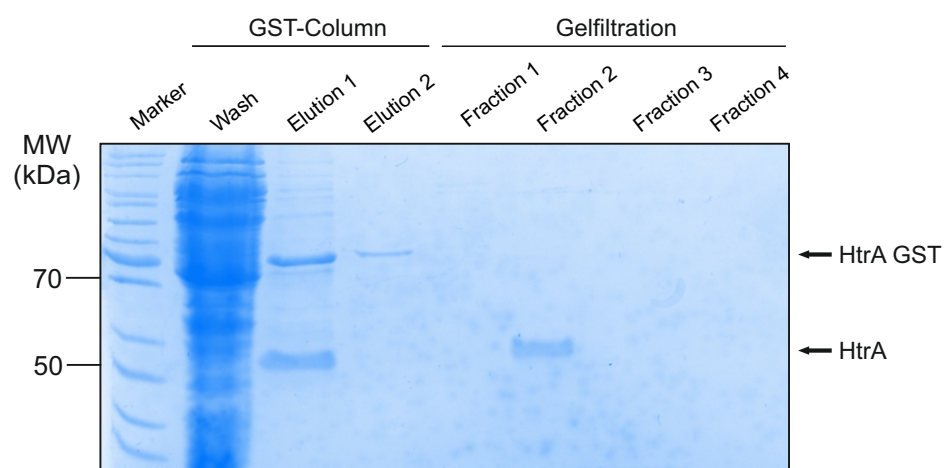

Supplement: Supplementary file 1 — Additional file 1: Figure S1. Purification of recombinant C. jejuni HtrA expressed in E. coli BL21. Important purification steps are shown, including purification by use of a GST-affinity column, exemplary wash and two elution fractions. The final purification step was performed via gel filtration, showing purified HtrA in fraction 2. [file 13099_2019_295_MOESM1_ESM.pdf]
